# Motion Direction Discrimination with Tactile Random-Dot Kinematograms

**DOI:** 10.1177/20416695211004620

**Published:** 2021-03-28

**Authors:** Scinob Kuroki, Shin’ya Nishida

**Affiliations:** NTT Communication Science Laboratories, NTT Corporation, Kanagawa, Japan; NTT Communication Science Laboratories, NTT Corporation, Kanagawa, Japan; Department of Intelligence Science and Technology, Graduate School of Informatics, Kyoto University, Kyoto, Japan

**Keywords:** haptics, apparent motion, random-dot kinematogram, motion, direction

## Abstract

Motion detection is a fundamental sensory function for multiple modalities, including touch, but the mechanisms underlying tactile motion detection are not well understood. While previous findings supported the existence of high-level feature tracking, it remains unclear whether there also exist low-level motion sensing that directly detects a local spatio-temporal correlation in the skin-stimulation pattern. To elucidate this mechanism, we presented, on braille displays, tactile random-dot kinematograms, similar to those widely used in visual motion research, which enables us to independently manipulate feature trackability and various parameters of local motion. We found that a human observer is able to detect the direction of difficult-to-track tactile motions presented to the fingers and palms. In addition, the direction-discrimination performance was better when the stimuli were presented along the fingers than when presented across the fingers. These results indicate that low-level motion sensing, in addition to high-level tracking, contribute to tactile motion perception.

## Introduction

Sensing movements of touched objects on the surface of fingers must be useful for us humans to properly interact with the environment, for example, as a guide for moving the hand and as a slip detector to prevent dropping objects. Despite its functional importance, the computational and neural mechanism of human tactile motion perception from cutaneous inputs remains poorly understood. We have been addressing this problem by psychophysically analysing various aspects of haptic motion perception (Kuroki & Nishida, 2018; Kuroki et al., 2011, 2016), and this study is a part of this endeavour.

Given that object movement is defined as a change in the object location across time, one natural computation to estimate the motion signal is to identify the location of the object then track it over time. Alternatively, without explicitly specifying the object location, the sensory system can directly compute the motion signal from a local spatio-temporal correlation, spatio-temporal slant, or motion energy of the input pattern. A large body of literature suggested the contribution of both types of motion computation to human motion perception, at least for vision (see review for Nishida, 2011). Previous vision studies have called the latter mechanism short-range (Braddick, 1974; Chubb & Sperling, 1989) or first-order motion (Adelson & Bergen, 1985), while the former, long-range or third-order motion. To highlight the computational difference, we call the two types of motion computation high-level feature tracking and low-level motion sensing, respectively.

The contribution of low-level motion sensing to tactile motion perception remains controversial. Although physiological studies suggest that some neurons in the primary somatosensory cortex may respond to low-level motion signals (DiCarlo & Johnson, 2000; Essick & Whitsel, 1988; Pack & Bensmaia, 2015; Pei et al., 2010), *behavioural* evidence is sparse. Behavioural studies rather favour the idea that tactile motion is dominantly processed by higher level tracking. For example, it has been reported that participants described the perceived direction as surprisingly uncertain when they touched an unambiguously rotating cylinder, which was not the case when they saw a similar stimulus (Holcombe & Seizova-Cajic, 2008). In our previous studies (Kuroki & Nishida, 2018; Kuroki et al., 2016), we investigated the tactile discrimination of two (or three) asynchronous sine waves (considerably containing low-level motion energy components) from those of synchronous sine waves (containing no motion components) and found that the task was nearly impossible with across-finger (long-range) stimulus presentation (Kuroki et al., 2016) but well above chance with within-finger (short-range) presentation (Kuroki & Nishida, 2018). While the latter finding supports the ability to detect motion energy within small somatotopic areas, discrimination of phase-shift direction (motion energy shift direction) was found to be very difficult (Kuroki & Nishida, 2018). Another line of studies used motion aftereffects to investigate underlying tactile-motion processing. In touch, the motion aftereffects measured with ambiguous test stimuli have been extensively reported, but the occurrence of the motion aftereffects with stationary test stimuli remains obscure (Konkle et al., 2009; Kuroki et al., 2011; Planetta & Servos, 2008; Watanabe et al., 2007). In vision, it is considered that the motion aftereffects reflect multiple levels of processing, but that the use of stationary test stimuli is a means of probing low-level motion processing (Nishida & Ashida, 2000). If vision-touch analogy works, the existence of low-level motion sensing in touch is not supported by the reports of tactile motion aftereffects. Indeed, the tactile motion aftereffects measured with ambiguous test stimuli occur in the spatiotopic coordinate rather than in the somatotopic coordinate (Kuroki et al., 2011), which can be driven even by visual motion stimuli (Konkle et al., 2009), and show minor indication of speed tuning (McIntyre, Birznieks, et al., 2016; McIntyre, Seizova-Cajic, et al., 2016). These findings indicated the contribution of higher level, even super-modal, mechanisms to tactile motion aftereffects.

One reason behind the shortage of evidence for low-level motion sensing might be the limitation of the used stimuli. Most previous tactile-motion studies used easily traceable stimuli, such as regular patterns and simple shapes, moving at a constant rate (Amemiya et al., 2017; Bensmaïa et al., 2006; Craig, 2003; Holcombe & Seizova-Cajic, 2008; Seizova-Cajic & Taylor, 2014). Some used array stimulators (Gardner & Sklar, 1994; Konkle et al., 2009; McIntyre, Birznieks, et al., 2016; McIntyre, Seizova-Cajic, et al., 2016; Pei & Bensmaia, 2014), however, presented stimuli containing rich trackable features; thus, it is not easy to examine the contributions of low-level motion sensing separately from those of high-level tracking. To overcome this limitation, here we use tactile random-dot kinematogram (t-RDK) stimuli that enable easy control of trackability. The stimuli were presented on braille displays with more than 500 independent tactile actuators.

Visual RDKs have been widely used to investigate the visual motion processing in the brain. One of the advantages of using RDKs is density controllability. (When translated into touch, density is the number of possible points of contact on the skin.) With dense stimuli, trackable features are masked; thus, high-level feature tracking becomes difficult. In other words, increasing dot density creates a bias towards low-level motion sensing (Braddick, 1974). Another advantage is lifetime controllability. Lifetime is defined as the number of successive image frames in which each dot appears and moves before it extinguishes. (When translated into touch, lifetime is the number of individual actuators successively representing the same moving dot at different times.) With long/infinite lifetime RDKs, not only individual dots but also the global configurational features defined by dot clouds (e.g., centre of gravity) continuously shift in one direction for a while. Motion trajectories of dots are uniform and easy to track. Thus, long/infinite lifetime RDKs may be detected by high-level feature tracking in addition to low-level motion sensing. With short-lifetime RDKs, on the other hand, while local motion cues of individual dots persist for a few frames, global dot configuration continuously changes over time, which makes global motion difficult to track. Thus, decreasing lifetime creates a condition favouring low-level motion sensing. In addition, tracking may become difficult with fast and dense stimuli because of the limitation of attention (Lu & Sperling, 1995).

Although our t-RDKs were not as large and dense as standard visual RDKs due to the limitation of the presentation device, we expect that the basic effects of critical stimulus parameters, such as density, lifetime, and speed, on the relative contributions of low-level and high-level motion mechanisms should be similar between our t-RDKs and visual RDKs, and, as far as we are aware, there is no strong reason against this expectation.

We present tactile motion stimuli to fingers in which the direction of motion relative to the axis of the fingers may be another stimulus manipulation that affects the relative contributions of the low- and high-level motion mechanisms. When continuous motion is presented along the axis of the fingers, the motion trajectory is almost always within the fingers, while when the motion is presented orthogonal to the axis of the fingers, the motion path runs across the fingers. We recently found that motion detection differs between within- and inter-finger motions (Kuroki & Nishida, 2018). When continuous low-frequency sine-wave vibrations were presented on finger(s) with a phase difference, the performance of asynchrony detection of the stimuli (which is likely to be related to low-level motion sensing) was significantly higher than the chance level when the stimuli were presented within one finger but not across neighbouring fingers. For this study, therefore, we conducted t-RDK experiments with two directions of motion: motion along the long axis of the fingers that predominantly taps into within-finger motion detectors and motion along the short axis of fingers that taps inter-finger motion detectors in addition to within-finger ones.

Based on the knowledge gathered from visual RDK studies, we hypothesised that if a tactile motion system relies solely on high-level feature tracking, the direction detection of t-RDKs will be severely impaired with dense, short-lifetime, and fast stimuli. This will not be the case if low-level motion sensing also occurs. Experiment 1 involved testing the direction-discrimination performance by presenting t-RDKs on fingers. We varied dot lifetime, density, speed, and moving direction. For comparison, we additionally measured the visual-discrimination performance with our RDK stimuli. As this experiment showed a clear difference in the performance of tactile direction discrimination between motion direction along the long axis of the fingers (i.e., vertical, completely within-finger motion) and motion direction along the short axis (i.e., horizontal, partially across-finger motion), Experiment 2 involved exploring this issue in depth. As the dots presented between fingers could not be touched, there remained possibility that motion along the short axis was interrupted. To mimic this interruption with motion along the long axis, we tested control condition wherein the dots were partially occluded by mask stimuli. Experiment 3 involved changing not only the number but also the size of the dot stimuli as another potential parameter to change the contribution of low-level and high-level motion mechanisms. To test the generality of our findings, Experiment 4 involved presenting t-RDKs on a different body part, the palm. Experiment 5 involved presenting t-RDKs on a fingertip. As the stimulus area was small in this condition, we were able to use a stimulator with a high refresh rate than that used in other experiments. So we also investigated the direction-discrimination performance with fast-moving dots.

We found the direction-discrimination performance with dense or short-lifetime t-RDKs was significantly above the chance level, while not as good for the same task done with vision. This detection performance was also better for motion along the long axis of the fingers than for motion along the short axis. This performance anisotropy between vertical and horizontal motion detection was minor when the stimuli became trackable or were presented within a single palm/finger. Our findings are consistent with the notion that in addition to high-level feature-tracking, low-level motion sensing plays a role in tactile motion perception.

## Methods

### Participants

One of the authors (S. K.) and 26 volunteers (15 females), aged 21 to 42 and all right-handed, participated in the five experiments we conducted. Ten participated in each experiment, with partial overlaps of participants across the five experiments. They gave written informed consent before the start of the experiments. The volunteers had no specialised knowledge of psychophysical experiments and were unaware of the purpose of the experiments. Recruitment of participants and experimental procedures were approved by the NTT Communication Science Laboratory Research Ethics Committee and were conducted in accordance with the Declaration of Helsinki.

### Apparatus

An arrayed piezoelectric *braille* stimulator (Dot View DV-2; KGS Corp., Japan) was used to deliver t-RDKs in the main experiments. Each pin can independently take either the on position (maximum of 0.7 mm, less when damped by the contacting hand) or off position at a refresh rate of 10 Hz. Note that on-pins did not vibrate (which is typical of other braille systems such as OPTACON; Telesensory Systems, Palo Alto, CA) but remained stationary during the specified period with a rise time of 15 milliseconds. The number of pins changed depending on the condition. In Experiments 1–3, stimuli were presented using a 26 × 26 array of pins (1.3 mm in diameter, 2.4-mm inter-pin distance), to a 61.3 × 61.3 mm area of index, middle, ring, and little fingers. In Experiment 4, stimuli were presented using a 26 × 38 array of pins, to a 61.3 × 90.1 mm area of a palm (proximal–distal axis [side depth] was shorter than radial–ulnar axis [front width]). In Experiment 5, stimuli were presented using a 6 × 6 array of pins to a 13.3 × 13.3 mm area of an index or middle finger. In addition to the stimulator that we used in the first four experiments (Dot View DV-2), we also used a different type of braille stimulator (TI-1101, KGS Corp., Japan), which was smaller (2 × 4 array of pins, covering 3.7 × 8.5 mm) but faster (refresh rate of 128 Hz with a rise time of 3 milliseconds). The pin size was the same, but on-pins were vibrated at 128 pulses per second.

A participant sat at a table with their right or left finger(s)/palm placed on the stimulator. The stimulator was located to the right/left of the body midline when the participant used their right/left hand so that they could comfortably place their finger(s)/palm. The stimulator always contacted the skin throughout the experiment. Participants responded by pressing a keyboard with their free hands. They performed the tasks with their eyes open to maintain their arousal level, but the moving parts of stimulators were occluded by a black board from the participants’ view. Under the vision condition, the occluder was removed and participants’ hands/finger(s) were not placed on the stimulator so that the participants could see the stimuli. They wore earplugs to mask noise produced by the stimulator.

### Stimulus Design

We changed stimulus density, speed, and lifetime. The duration of the stimulus was always 1,000 milliseconds. The initial location of on-pins was randomly chosen, and the number of on-pins was based on density. Motion stimuli were produced by shifting the location of on-pins in one direction either upward or downward under vertical motion condition, and the direction was randomly determined for each trial. Under horizontal motion condition, motion direction was either rightward or leftward.

With the main stimulator (Dot View DV-2), motion speed was set by how long the on-pins stayed at the same location before moving and the number of gaps (off-pins) between the current and next locations of the activated on-pins. For example, to present motion at 24 mm/second, pins in the neighbouring column were successively activated every 100 milliseconds. To present motion at 48 mm/second, pins in every other column (i.e., one gap) were successively activated every 100 milliseconds. To present motion at 4.8 mm/second, pins in the neighbouring column were successively activated only once at the middle of the 1000-millisecond stimulus duration (this extreme situation is for explanation, and we did not actually present this very slow motion.). A small and fast stimulator (TI-1101) was used in addition to the one used in the other experiments in Experiment 5, where motion speed was set only by changing the duration of the on-pins staying stationary before moving. This stimulator has a high refresh rate (128 Hz) and can present motion without a gap between the current and next on-pins. Note that this stimulator can produce t-RDKs only along its long axis as there are only 2 × 4 arrays of pins. Thus, the stimulator was placed vertically under the vertical-motion condition while placed horizontally under horizontal-motion condition.

**Figure 1.**
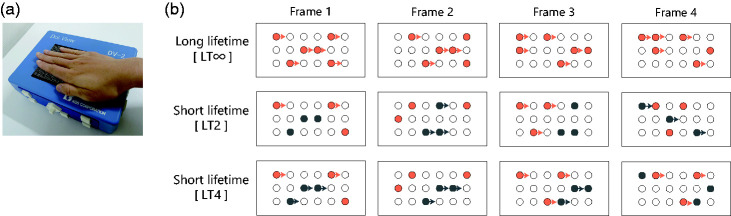
a: Braille-type stimulator. b: Schematic representations of pin array and activation patterns with rightward motion under three different lifetime conditions. Filled circles indicate on (activated) pins. Top row illustrates the LT∞ where all on-pins except those overflowing at the right boundary of stimulus area kept moving rightwards. Middle row illustrates the LT2. Pins belonging to orange group randomly changed locations at every odd (2*n* + 1, *n* denotes natural number) frame and moved rightwards from odd to even (2*n*) frames, while those belonging to grey group changed locations at every even frame and moved rightwards from even to odd frames. Bottom row illustrates the LT4 where pins belonging to orange group changed locations at every 4*n* + 3 frames, while those belonging to grey group changed locations at every 4*n* + 1 frames. LT∞: lifetime-infinite condition; LT2: lifetime 2 condition; LT4: lifetime 4 condition.

Under the lifetime-infinite condition (LT∞), all the on-pins coherently moved (their positions were shifted by the same distance in the same direction) between frames. Thus, the global patterns of the on-pins were kept except those of pins overflowing at the boundary of the stimulus area (Figure 1b). When an on-pin reached the boundary, it reappeared at the other boundary. This loop structure gave a continuous impression of unidirectional motion across the stimulus area. Under the lifetime 2 condition (LT2), on-pins were divided into two groups (orange and grey in Figure 1b middle row). In the first frame, all the on-pins appeared at random locations. In the second frame, the first group (orange pins) moved in the designated direction, while the other group (grey) appeared at new locations. In the third frame, the first group appeared at new locations, while the second group moved. This pattern was repeated until the end of stimulus presentation. As each pin movement lasted for two frames, they are called lifetime 2 stimuli. Under the lifetime 4 condition (LT4), on-pins were again divided into two groups. In the first frame, all the on-pins appeared at random positions. In the second frame, they all moved. In the third frame, the first group of pins (orange) appeared at new random locations, while the second group (grey) moved. In the fourth frame, all the pins moved. In the fifth frame, the first group of pins (orange) moved, while the second group (grey) appeared at new random locations. This pattern was repeated until the end of stimulus presentation.

Under the mask condition in Experiment 2, static lines (in which pins were always on) were presented in addition to the vertically moving t-RDK stimuli. This is the condition inspired by slit vision of visual experiments. As these lines/masks partially occluded the t-RDK stimuli, the participants could feel the vertical motion only in the area without the masks. Each mask consisted of a 2 × 26 array of on-pins, and inter-mask distances were six pins (13.3 mm).

In Experiment 3, the locations of on-pins were grouped as if an t-RDK consisted of large moving dots. Under this large (grouped) dot condition, each dot consisted of 3 × 3 arrays of on-pins; while under the small (scattered) dot condition, each dot consisted of one pin.

### Procedure

At the beginning of each trial, the braille stimulator presented an outline box to remind the participant of the boundary of the presentation area of the t-RDK stimuli. After 1,000 milliseconds, all pins took the off position and the t-RDK stimuli started after a 500-millisecond blank period. After presenting stimuli for 1,000 milliseconds, all pins took the off position. Participants made a two-alternative forced choice as to whether the motion was upward or downward under vertical condition or rightward or leftward under horizontal condition. After each response, a feedback signal was presented to a participant by sound to maximise their task performance. They evenly used their right and left hands and were told not to move their hands on the stimulator during one session, which lasted less than 5 minutes.

### Experiment 1: Effect of Motion Direction of t-RDKs on Fingers

Participants touched the t-RDK stimuli moving vertically or horizontally (i.e., along the long or short axis) with their fingers or watched the stimuli moving vertically. Each participant performed 2,700 trials (20 Repeats × 3 Conditions [vertical vision, vertical tactile, horizontal tactile] × 5 Speeds [15, 31, 46, 92, 184 mm/second] × 3 Densities [4, 20, 100 dots] × 3 Lifetimes [∞, 4, 2]). Note that the chosen speed was [0.25, 0.5, 0.75, 1.5, 3.0] the length of the stimulus area.

### Experiment 2: Effect of Motion Direction of t-RDKs on Fingers w/wo Masks

Participants again touched the t-RDK stimuli with their fingers. There were three stimulus conditions: horizontally moving t-RDKs, vertically moving t-RDKs, and vertically moving t-RDKs with masks (three static lines). Each participant performed 1,440 trials (20 Repeats × 3 Conditions [horizontal, vertical, vertical with masks] × 4 Speeds [15, 31, 46, 92 mm/second] × 3 Densities [4, 20, 100 dots] × 2 Lifetimes [∞, 4]).

### Experiment 3: Effect of Size of Dot of t-RDKs on Fingers

Participants touched the t-RDK stimuli with their fingers. There was a total of four conditions, two densities per two dot sizes (large dots: nine pins or small dots: one pin). Each participant performed 1,920 trials (20 Repeats × 4 Conditions [large dots with vertical motion, large dots with horizontal motion, small dots with vertical motion, small dots with horizontal motion] × 4 Speeds [15, 31, 46, 92 mm/second] × 2 Densities [4, 10 dots for large-dot condition, 4, 100 dots for small-dot condition] × 3 Lifetimes [∞, 4, 2]).

### Experiment 4: Effect of Motion Direction of t-RDKs on Palm

Participants touched the t-RDK stimuli with their palms. The stimuli were presented over a wider area than they were presented for the fingers. Each participant performed 1,800 trials (20 Repeats × 2 Directions [vertical or horizontal] × 5 Speeds [9.0, 45, 90, 180, 270 mm/second] × 3 Densities [4, 20, 100 dots] × 3 Lifetimes [∞, 4, 2]).

### Experiment 5: Effect of Motion Direction of t-RDKs on Fingertip

Participants touched the t-RDK stimuli with their index or middle finger. There were two stimulator conditions (6 × 6 pins of slow stimulator, 2 × 4 pins of fast stimulator). Each participant performed 2080 trials (20 Repeats × 2 Direction [vertical or horizontal] × 4 Speeds [6.0, 12, 24, 48 mm/second] × 2 Densities [1, 4 dots] × 3 Lifetimes [∞, 4, 2] with slow stimulator; 20 Repeats × 2 Directions [vertical or horizontal] × 7 Speeds [4.8, 9.6, 19, 38, 77, 154, 307 mm/second] × 2 Densities [1, 2 dots] × 2 Lifetimes [∞, 2] with fast stimulator).

### Data Analysis

The data consisted of each participant’s directional judgements (up or down for vertical motion, left or right for horizontal motion) for each stimulus with a specific combination of direction, lifetime, number of dots, and speed. Under each stimulus condition, the proportion-correct score for each individual was first calculated, and then the overall mean across all participants and an uncorrected 95% confidence interval (CI) were calculated.

To evaluate the differences across conditions, after transferring the proportion-correct scores to the Z scores using the inverse of the cumulative normal distribution function, we conducted repeated measures analysis of variance (ANOVA). When necessary, *p* values in post-hoc tests were corrected according to Shaffer’s (1986) modified sequentially rejective Bonferroni procedure.

In Experiment 1, the correlation between the directional detection of vertical motion by touch and that by vision was calculated using the raw data of individual participants for each combination of stimulus conditions.

## Results

### Experiment 1: Effect of Motion Direction of t-RDKs on Fingers

**Figure 2.**
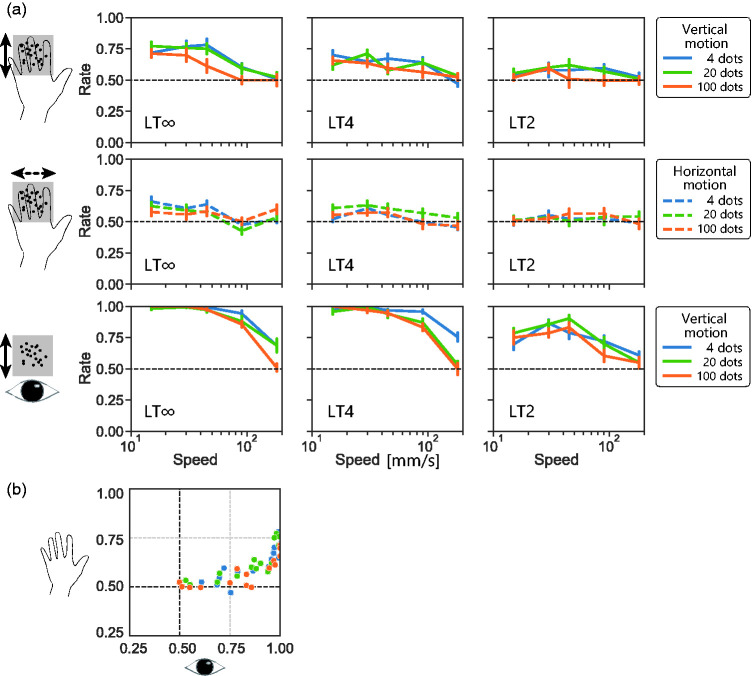
Results from Experiment 1. a: Mean correct rates of two-alternative direction discrimination of t-RDK stimuli as function of stimulus speed. Error bars represent uncorrected 95% confidence interval. Colours (blue, green, and orange) represent presented number of dots (4, 20, and 100). Panels in left, middle, and right columns represent results of LT∞, LT4, and LT2 t-RDKs. Panels in top and middle rows represent results of touch condition where participants put their hands (fingers) over braille and haptically observed t-RDKs without moving their hands. Three panels in the bottom row represent results under vision condition where participants visually observed RDKs made by pin movements on braille stimulator. b: Scatter plot showing relationship between direction-discrimination performances under touch (top row in (a)) and vision (bottom row in (a)) conditions. Each dot represents mean correct rate of each combination of density, lifetime, and speed. Colours represent presented number of dots. LT∞: lifetime-infinite condition; LT2: lifetime 2 condition; LT4: lifetime 4 condition.

In Experiment 1, we tested whether the direction of dense, short, and fast t-RDK stimuli can be haptically detected. The results with vertical (distal-proximal) motion are shown in the top row of Figure 2, where panel and colour represent lifetime and number of moving dots, respectively. All three factors—stimulus density, lifetime, and speed—appeared to influence the detection of motion direction: denser, shorter, or faster stimuli made motion direction discrimination more difficult. A three-way ANOVA indicated significant main effects for all three factors, *F*(2, 0.88) = 5.8, *p* < .03, η^2^ = 0.019 for density, *F*(2, 4.2) = 23, *p* < .0001, η^2^ = 0.089 for lifetime, *F*(4, 3.1) = 13, *p* < .0001, η^2^ = 0.13 for speed. Importantly, however, the direction-discrimination performance was generally above the chance level (0.5 as our task was two-alternative forced choice) unless all three difficult conditions were combined (i.e., dense, short, and fast condition). The top left panel represents the results under LT∞ (i.e., continuous motion) stimuli. Under all density conditions represented in different coloured lines, the direction-detection performance was above the chance level for slow stimuli. The densest stimulus condition (100 dots represented with orange lines) resulted in the lowest performance, while the other two conditions resulted in similar performance (i.e., 95% CIs of blue and green lines overlapped). This trend was observed across different lifetime conditions, with performance dropping with shorter lifetime. With the shortest lifetime stimuli (LT2, top right panel) where each dot disappeared right after one hopping from the initial location, the performance was not significantly above the chance level under the fast and dense conditions.

The performance with horizontal (ulnar–radial) t-RDKs (middle row of Figure 2) was in general lower than that with vertical (distal-proximal) t-RDKs, *F*(1, 7.6) = 28, *p* < .001, η^2^ = 0.053. CIs never reached 0.75 under any condition even under LT∞ (middle left panel). This discrepancy between motion directions was further investigated in Experiment 2.

As our tactile display had a low refresh rate (10 Hz), high-speed motion stimuli with a large jump size may produce ambiguous motion. Thus, there remains a possibility that the observed performance drop at high speeds simply reflects the limitation of our display. To check this, we asked participants to remove their hands, look directly at the tactile display, and report the direction of the RDKs. The results under the visual condition (bottom three panels of Figure 2a) also showed performance drop with increasing speed. This suggests that our display produced reliable directional signals even at high speeds.

We also found a high correlation in direction-discrimination performance between touch (vertical motion condition) and vision (Pearson’s *r* = .79, *p* = 9.8), though the absolute performance level was much higher for vision. This suggests a qualitative similarity in motion processing between the two modalities.

In summary, we observed that the direction of dense and short t-RDK stimuli can be detected. This detection performance was quantitatively much lower, but qualitatively similar, when compared with the direction-discrimination performance in the same task done by eyes. We also observed a large performance asymmetry between vertical along-finger motion and horizontal across-finger motion; the former was easy to detect while the latter was difficult. The direction-discrimination performance dropped as speed increased. The effect of stimulus speed was further investigated in Experiment 5.

### Experiment 2: Effect of Motion Direction of t-RDKs on Fingers w/wo Masks

**Figure 3. fig3-20416695211004620:**
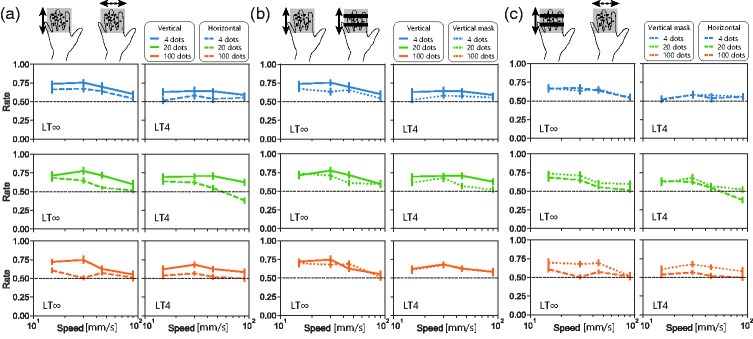
Results from Experiment 2. a: Direction discrimination with vertically and horizontally moving t-RDKs without masks. b: Direction discrimination under vertical motion with and without masks. c: Direction detection under vertical motion with masks and horizontal motion without masks. For readability, each panel compares results of two out of the three conditions. LT∞: lifetime-infinite condition; LT4: lifetime 4 condition.

There is a physical difference between along-finger motion and across-finger motion, that is, the gap and discontinuation of the motion. As our fingers have pole-like shapes while braille has a flat surface, dots presented between fingers cannot be touched nor detected. This causes information loss under both conditions, but motion interruption may be severer under the across-finger condition. To mimic this motion interruption under the along-finger condition, we occluded moving dots by presenting physical masks (made from stationary on-pins) in the orientation orthogonal to the motion direction. There were three conditions in this experiment: vertical motion without masks, vertical motion with masks, and horizontal motion without masks. ANOVA revealed a significant main effect of the conditions, *F*(2, 5.1) = 15, *p* < .001, η^2^ = 0.077. A post-hoc test (*p* < .05) showed that the direction-discrimination performance under vertical (along finger) motion was better than that under horizontal (across finger) motion (*p* < .01; Figure 3a), which is consistent with the results from Experiment 1. Due to the disruption effect of masks, the performance under vertical mask condition was lower than that under vertical without mask condition as expected (*p* < .01; Figure 3b). What is more, the performance under along-finger motion condition was still better than that under the across-finger motion condition even with masks (*p* = .02; Figure 3c). Thus, performance difference according to motion direction observed in Experiment 1 cannot be simply explained by the effect of the physical occlusion of the dots. In addition, the interaction of the Main Condition × Density indicated that the pattern of change in performance differs between that due to the existence of the masks and that due to the stimulus orientation, *F*(4, 0.52) = 4.7, *p* < .01, η^2^ = 0.016. The direction-discrimination performance drop under vertical motion condition due to masks (Figure 3b) was apparent when t-RDK was composed of a smaller number of dots (multiple comparison for conditions at dot = 4, *p* < .01; dot = 20, *p* < .01), but not when the number of dots was large (dot = 100, *p* = .84). This is reasonable as even when a significant portion of the stimulus area was occluded, local motion signals sufficient to perform the task were more likely to remain under the dense condition than under the sparse condition. On the other hand, performance discrepancy between vertical and horizontal motion conditions was obvious when the number of dots was large (vertical vs. horizontal in Figure 3a orange, *p* < .01; vertical-mask vs. horizontal in Figure 3c orange, *p* < .01), while not when the number was small (vertical vs. horizontal in Figure 3a blue, *p* = .96; vertical-mask vs. horizontal in Figure 3c blue, *p* = .03). Thus, the performance difference due to motion direction cannot be ascribed solely to the gap and discontinuation of the motion.

### Experiment 3: Effect of Size of Dot of t-RDKs on Fingers

**Figure 4. fig4-20416695211004620:**
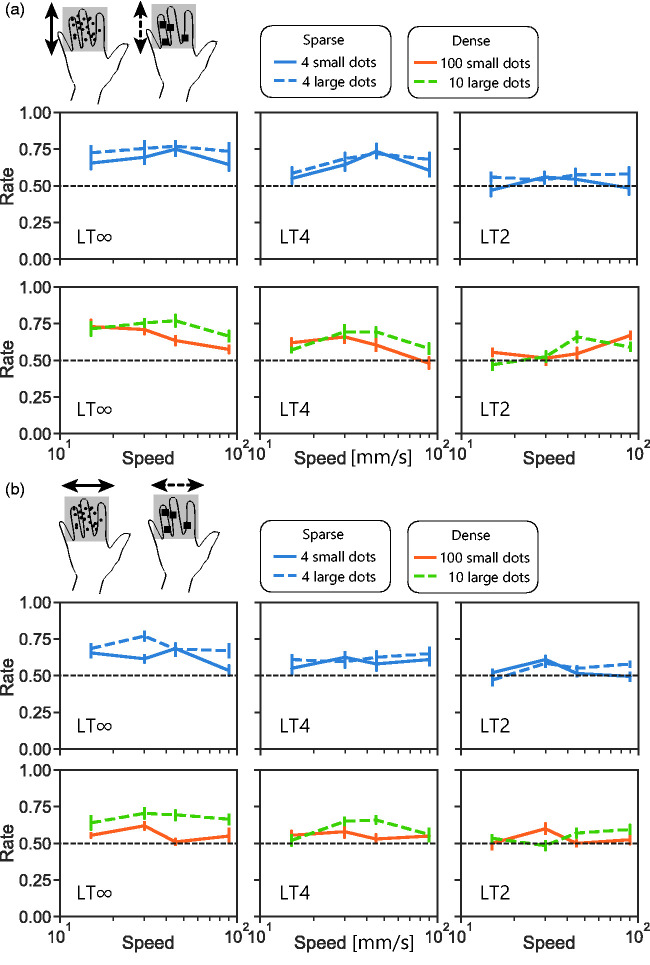
Results from Experiment 3. a: Direction detection with vertically moving t-RDKs with small dots and large dots. Under small dots condition, each dot consisted of one pin, as in Experiments 1–5. Under large dots condition, each dot consisted of nine pins. b: Direction detection with horizontally moving t-RDKs. LT∞: lifetime-infinite condition; LT2: lifetime 2 condition; LT4: lifetime 4 condition.

In visual studies using RDKs, a shift from low-level processing to high-level processing has been suggested to occur not only with decreasing dot number but also with increasing dot size (Nishida & Sato, 1995; Sato, 1998; Smith & Ledgeway, 2001). We examined whether the direction-discrimination performance with t-RDKs, especially asymmetry in direction, changes according to dot size. In this experiment, we tested two different sized dots; large dots were 9 times larger than the original small dots. Overall, the performance with large dots (Figure 4 dashed line) was better than with small dots (Figure 4 solid line), *F*(1, 4.3) = 27, *p* < .001, η^2^ = 0.026. With large dots, where high-level processing was assumed to be working, the performance was well above the chance level even under horizontal motion. When directly comparing the performance with four dots of different sizes (Figure 4 blue), performance with the large dots was higher than that with the small dots, *F*(1, 2.2) = 26, *p* < .001, η^2^ = 0.023, although the number of on-pins (i.e., energy) was also higher with the former. When directly comparing the performance of 10 gathered large dots and that of 100 scattered small dots (Figure 4 orange and green), the former was higher than the latter even though the number of on-pins was nearly the same, *F*(1, 2.1) = 17, *p* < .01, η^2^ = 0.031.

### Experiment 4: Effect of Motion Direction of t-RDKs on Palm

**Figure 5. fig5-20416695211004620:**
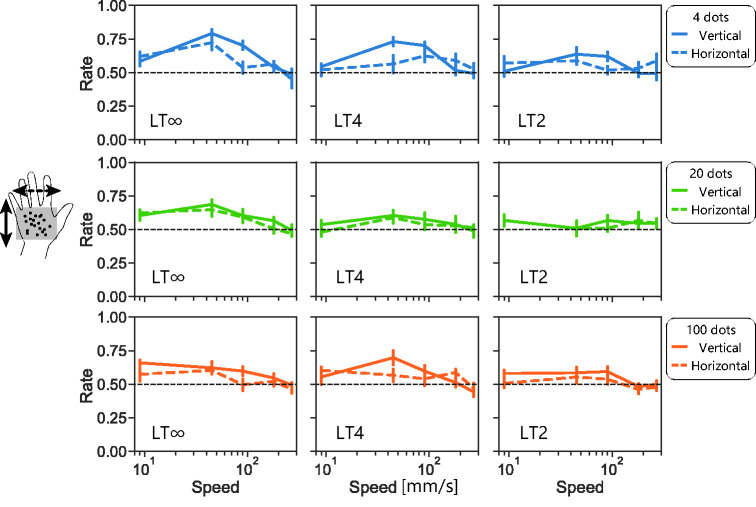
Results from Experiment 4. Direction discrimination with vertically and horizontally moving t-RDKs for palm. Note that t-RDKs were presented with wider area (26 × 38 array, longer in radial–ulnar axis) in this experiment, compared with those in Experiments 1–3 (26 × 26 array), where t-RDKs were presented to fingers. LT∞: lifetime-infinite condition; LT2: lifetime 2 condition; LT4: lifetime 4 condition.

In this experiment, we changed the stimulation area of the hand and investigated whether the direction of dense and short-lifetime t-RDK stimuli can be detected with palm. As a result, the direction-discrimination performance above the chance level was observed (Figure 5) and the average peak performance was around 0.75, which is comparable to those obtained with fingers (Experiments 1 and 2). In this experiment, both vertical and horizontal motions could be detected with the palm, and the performance asymmetry became small: ANOVA revealed no significant main effect of the stimulus direction (*p* > .1).

### Experiment 5: Effect of Motion Direction of t-RDKs on Fingertip

**Figure 6.**
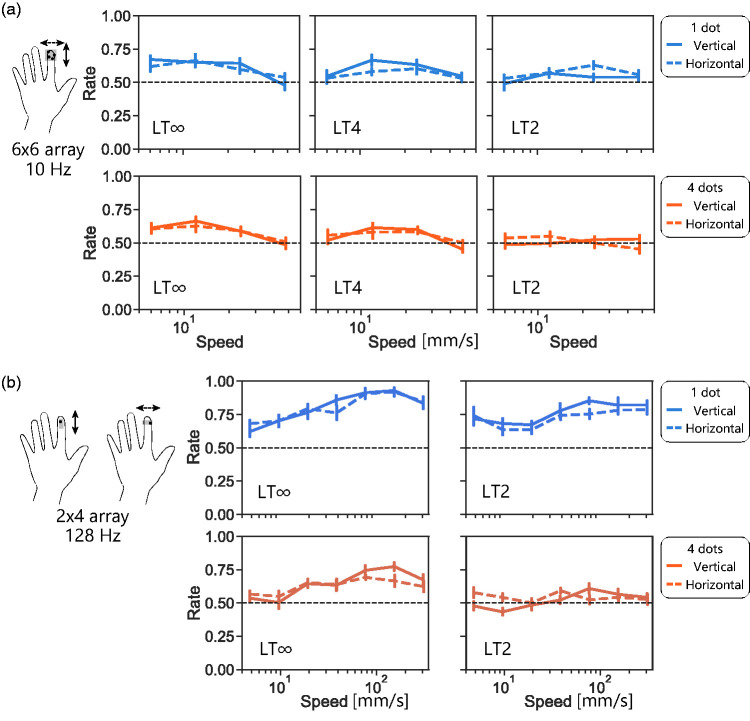
Results from Experiment 5. Direction discrimination with vertically and horizontally moving t-RDKs for fingertip. a: t-RDK stimuli were presented with slow and large braille (Dot View DV-2: 6 × 6 array with refresh rate of 10 Hz), which was used in other experiments. b: t-RDK stimuli were presented with fast and small braille (TI-1101: 2 × 4 array with refresh rate of 128 Hz). Here, LT4 stimuli are very close to LT∞ stimuli due to limited number of arrays (4); thus, we only tested LT2 and LT∞ conditions. LT∞: lifetime-infinite condition; LT2: lifetime 2 condition; LT4: lifetime 4 condition.

Finally, we presented t-RDKs to the fingertip of the index finger. Because of the limitation of the size of the fingertip, we could only test with small pin numbers (one or four dots with 6 × 6 array). Again, the direction-discrimination performance above the chance level was observed (Figure 6a), and the average peak performance was around 0.7, which is slightly lower than those obtained with fingers and palm (Experiments 1, 2, and 4). The direction-discrimination performance was slightly lower under the dense condition (4 in 36 pins, 11% of the total pins were on) than under the sparse condition (1 in 36 pins, 0.028%), but the difference was not statistically significant, *F*(1, 1.0) = 4.2, *p* = .071, η^2^ = 0.02. The performance under the shorter lifetime condition was lower than that under the longer lifetime condition, *F*(1, 1.3) = 20, *p* < .0001, η^2^ = 0.048, and directional asymmetry was not statistically significant (*p* > .1).

One advantage of this fingertip condition was that we could also use a different braille display that has a smaller array but with a high refresh rate (one or four dots with 2 × 4 array). We found that the overall direction-discrimination performance obtained with this display was higher (best score: 0.93) than that obtained with the original low refresh rate display (best score: 0.67; Figure 6b, note also the difference in the speed range between 6a and 6b). Moreover, the performance stayed above the chance level (0.5) even when the moving speed was very high (over 300 mm/second) except for the toughest condition (four dots and LT2, right bottom panel). The effect of the stimulus density on direction-discrimination performance was clearly observed, *F*(1, 93) = 55, *p* < .0001, η^2^ = 0.22: direction-discrimination performance was always lower under the dense condition (four in eight pins, 50%) than under the sparse condition (one in eight pins, 12.5%). Similar to the results with the slower display, the direction-discrimination performance under the shorter lifetime condition was lower than that under the longer lifetime condition, *F*(1, 23) = 16, *p* = .0031, η^2^ = 0.053, and directional asymmetry was not statistically significant (*p* > .1).

## Discussion

To address whether low-level motion sensing, in addition to high-level feature tracking, contributes to tactile motion perception, we tested the direction-discrimination performance of t-RDK stimuli by changing stimulus lifetime, density, and speed. Given that dense and short-lifetime stimuli are difficult to track, if human observers can detect the direction of these stimuli, then they must have a motion-detection mechanism other than high-level tracking.

**Figure 7.**
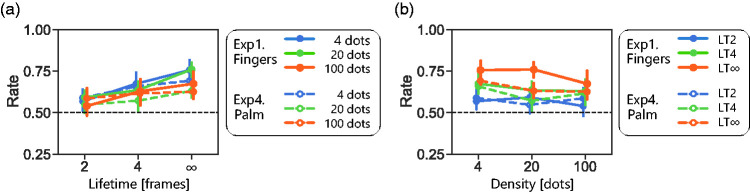
Direction-discrimination performances of vertically moving t-RDKs. Averaged performance at three slowest speeds was calculated as representative for each participant at each condition and then averaged across participants. Results are plotted as function of lifetime (a) and density (b) of stimuli. LT∞: lifetime-infinite condition; LT2: lifetime 2 condition; LT4: lifetime 4 condition.

Lifetime is a unique RDK parameter and defined as the number of successive image frames where stimuli appear and move before being extinguished. With normal (long lifetime) RDKs, motion direction can be computed from local motion cues as well as from position change of the global shape or the centre of gravity. Motion trajectories of dots are straight lines and easily calculated under this condition. With short-lifetime RDKs, on the other hand, local motion cues of individual dots remain, but most global cues are excluded. Thus, short-lifetime stimuli would be difficult to track by high-level feature tracking. In our experiments, we robustly observed higher direction-discrimination performance with long-lifetime t-RDKs, compared with that with short-lifetime ones (in general, the performance in the left columns was better compared with that in the right columns in Figures 2 [Fig fig3-20416695211004620][Fig fig4-20416695211004620][Fig fig5-20416695211004620]to 6). As shown in Figure 7a, direction-discrimination performance improves with longer lifetime in general. This is consistent with the idea that high-level tracking has a major role in detecting tactile motion. We reaffirmed the same trend with visual direction discrimination with our stimuli: direction-discrimination performance improved with longer lifetime. Nevertheless, an important finding is that even with LT4 t-RDKs for which this tracking may not properly work, the direction-discrimination performance was above the chance level for fingers, palm, and a single finger (middle column of Figures 2, 5, 6a, and 7a). This is consistent with the existence of low-level motion sensing in addition to high-level tracking.

Density is defined as the signal-to-background ratio, or the number of on-pins in an RDK stimulus. When the density is low, the stimuli look like moving features and can be tracked by high-level motion mechanisms. When the density is high, the stimuli look like a moving texture. Texture images, which contain fewer salient features, would be difficult for high-level tracking. In our experiments, an increase in dot density slightly impaired direction discrimination in some cases (Figure 7b), but importantly, the performance was above the chance level even under dense dot conditions. Again, this is consistent with the existence of low-level motion sensing in addition to high-level tracking. One may speculate on the contribution of high-frequency inputs to the observed direction-discrimination performance of t-RDK, especially in Experiment 5 conducted with the fast braille. High-frequency inputs are known to contribute to texture segregation (Weber et al., 2013), where a motion signal may yield some cue. However, high-frequency inputs mainly tap mechanoreceptor afferent channels with a large receptive field and fast temporal characteristics, and it remains unclear how these channels detect the local energy shift in short-range motion. The decoding performance of the motion direction was low when only using the activity of high-frequency sensitive afferents, and combining responses from other afferents led to improved performance (Saal et al., 2017).

The effect size of stimulus density (i.e., loss of feature) on direction discrimination seems different according to the stimulus direction. The direction-discrimination performances with the horizontal/ulnar–radial across-finger motion stimuli were always lower than those with vertical/distal–proximal along-finger motion stimuli, and this trend seems more evident with dense stimuli (Figure 3). For instance, our participants could not discriminate direction well above the chance level under the dense stimuli condition when the stimuli moved horizontally (orange dotted line in Figures 2 [Fig fig3-20416695211004620]to 4). As dense stimuli must be challenging for high-level tracking, it can be speculated that detection of across-finger motion mainly relies on high-level tracking rather than low-level motion sensing. Our results also suggest that this asymmetry is not a simple outcome from motion interruption caused by information loss of untouched dots presented between fingers. We observed that along-finger dense motion could be detected better than across-finger dense motion even when the motion was partially occluded as if there were physical gaps along fingers (Figure 3b). We also tested this asymmetry using a different approach: increasing dot size. With large dots, the stimuli can cover multiple fingers; thus, the across-finger motion became seamless even where there were physical gaps. Under the large dot LT∞ condition, where high-level processing was assumed to work, the performance asymmetry by direction still remained but was smaller as the across-finger motion was detected above the chance level (Figure 4a). Also, the asymmetry by direction was smaller when the stimuli were presented on the palm or a single fingertip (Figures 5 and 6). In summary, these results are consistent with the idea presented in our previous study (Kuroki & Nishida, 2018) that low-level motion sensing works in addition to high-level tracking, and the working ratio of these two mechanisms may differ between across-finger motion and along-finger motion.

Although our findings that tactile direction-discrimination performance improved with long-lifetime and sparse stimuli support the conventional view that high-level feature tracking dominates in tactile direction judgements (Holcombe & Seizova-Cajic, 2008; Konkle et al., 2009; Kuroki et al., 2016; McIntyre, Birznieks, et al., 2016; McIntyre, Seizova-Cajic, et al., 2016), our finding that direction-discrimination ability was not excluded for short lifetime, or dense t-RDKs, suggests the additional contribution of low-level motion sensing. Furthermore, the following findings are difficult to explain solely from the feature-tracking mechanism. First, we observed a quantitative similarity between vision and touch in direction-discrimination performance with our RDK stimuli (Figure 2b). This might be too accidental if the tactile system completely lacks low-level motion-sensing mechanisms as the visual system mainly uses low-level motion sensing for RDK detection (Braddick, 1974; Nishida, 2011). Second, we observed a difference in direction-discrimination performance between vertical (within-finger) and horizontal (across finger) directions for both sparse and dense t-RDKs (Figure 3). Importantly, the mask simulating inter-finger gaps excluded the directional difference for sparse t-RDKs but not for dense t-RDKs. These findings are difficult to interpret without assuming the presence of tactile local motion sensors that are more effective for along-finger directions. Third, if we can assume that low-level motion sensing in touch has a higher temporal limit than high-level feature tracking as in vision (Lu & Sperling, 1995), the existence of low-level motion sensing is also supported by improved direction discrimination for dense and rapid t-RDKs by switching the display to a fast 128-Hz braille (Figure 6b).

There were limitations to this study. Speed is a well-known parameter to divide attentional tracking and low-level motion sensing. Unfortunately, we could not present dense fast stimuli because of the stimulator limitation. We presented a small number of fast stimuli and observed reasonable direction-discrimination performance (Figure 6b), and the results are consistent with the existence of low-level motion sensing. Whether and how direction is detected with dense fast-moving stimuli remains an intriguing open question, which could be tested with better stimulators in future. It should be mentioned that we used braille-type stimulators, and the stimuli were sparsely and vertically represented as apparent motion. In other words, our stimuli were not designed to induce a directional distribution of skin stretch. Skin stretch is an additional low-level cue to motion detection, which is different to the low-level motion sensing discussed in this study. In daily life, motion on the fingertip occurs with skin stretch, and the brain has a good reason to use this information. The effect of skin stretch on motion detection should be investigated with a different setup in the future.

One important previous study (Pei et al., 2010) used t-RDK-like stimuli in addition to regular dots and bar stimuli and recorded robust representation of motion direction at Area 1 regardless of stimulus type. Their stimuli were four or five dots presented within a small area (1 cm × 1 cm) to the finger pad; thus, similar to our Experiment 5 with a slow stimulator (6 × 6 pin array) with four pins LT∞. Although we did not modulate stimulus coherence for stimulus clarity limited by the number of arrays, they successfully controlled it and found that fine tuning declined dramatically by decreasing the coherence level. As motion coherence is the basic parameter that controls the intensity of the low-level motion signal, one can speculate that activities of these neurons reflect the outcome of tactile low-level motion sensing. It is an interesting direction for future study to test the performance change in horizontal and vertical direction discrimination with fingers by modulating the coherence of the stimuli.

Using common experimental stimuli in visual motion, we characterised the mechanisms of tactile motion perception. We found not only similarity but also discrepancy in availability of these mechanisms between modalities. (Note, however, that consistent vertical skin stretch along motion direction lacked in our experiments because of the choice of stimulators, and there remains possibility that observed poor performance of the low level motion sensing could be improved with the presence of skin stretch cue.) In touch, long-range feature tracking dominates short-range motion sensing in most cases, and the contribution of latter process is not always conspicuous. Although detecting local motion energy is simplest to calculate motion and dominantly used in vision, one can hypothesise that this rather automatic calculation is not necessarily the best solution for tactile motion systems in real-life situations. Given that the skin covers most of the body with changes in the perpendicular direction, detected motion vectors from different populations of mechanoreceptors would be far from uniform most of the time. The brain must select specific areas of the skin to focus on to detect a meaningful direction of movement. In other words, automatic calculation of motion signals all over the skin surface might be too much computational cost/time consuming. Even after focusing on a specific area, skin is elastic, which may cause ill-posed problems in finding a source direction. When one part of the skin is stretched, the surrounding area should shrink. The deformations of the different skin locations interfere with each other in a complex manner. In addition, the surfaces have complex shapes (e.g., fingerprints, joints). Due to the local motion interactions, it does not necessarily mean that the motion pattern on the skin surface is coherent, even if the skin touches one moving stimulus. In contrast, high-level mechanisms, such as location/feature tracking, may be suitable in these situations.

## Conclusion

We presented t-RDK stimuli on the hand and tested the direction-discrimination performance of the stimuli by changing stimulus lifetime, density, and speed. The results showed that human observers can discriminate the direction of stimuli with little trackable features, such as dense and short-lifetime stimuli. These findings are consistent with the notion that in addition to high-level feature-tracking, low-level motion sensing plays a role in tactile motion perception.
